# Risk factors for post-ICU red blood cell transfusion: a prospective study

**DOI:** 10.1186/cc5041

**Published:** 2006-09-11

**Authors:** Sophie Marque, Alain Cariou, Jean-Daniel Chiche, Vincent Olivier Mallet, Frédéric Pene, Jean-Paul Mira, Jean-François Dhainaut, Yann-Erick Claessens

**Affiliations:** 1 Medical Intensive Care Unit, Cochin Hospital, rue du Faubourg Saint-Jacques, F-75679 Paris Cedex 14, France; 2 Department of Emergency Medicine, Cochin Hospital, rue du Faubourg Saint-Jacques, F-75679 Paris Cedex 14, France

## Abstract

**Introduction:**

Factors predictive of the need for red blood cell (RBC) transfusion in the intensive care unit (ICU) have been identified, but risk factors for transfusion after ICU discharge are unknown. This study aims identifies risk factors for RBC transfusion after discharge from the ICU.

**Methods:**

A prospective, monocentric observational study was conducted over a 6-month period in a 24-bed medical ICU in a French university hospital. Between June and December 2003, 550 critically ill patients were consecutively enrolled in the study.

**Results:**

A total of 428 patients survived after treatment in the ICU; 47 (11% of the survivors, 8.5% of the whole population) required RBC transfusion within 7 days after ICU discharge. Admission for sepsis (odds ratio [OR] 341.60, 95% confidence interval [CI] 20.35–5734.51), presence of an underlying malignancy (OR 32.6, 95%CI 3.8–280.1), female sex (OR 5.4, 95% CI 1.2–24.9), Logistic Organ Dysfunction score at ICU discharge (OR 1.45, 95% CI 1.1–1.9) and age (OR 1.06, 95% CI 1.02–1.12) were independently associated with RBC transfusion after ICU stay. Haemoglobin level at discharge predicted the need for delayed RBC transfusion. Use of vasopressors (OR 0.01, 95%CI 0.001–0.17) and haemoglobin level at discharge from the ICU (OR 0.02, 95% CI 0.007–0.09; *P *< 0.001) were strong independent predictors of transfusion of RBC 1 week after ICU discharge.

**Conclusion:**

Sepsis, underlying conditions, unresolved organ failures and haemoglobin level at discharge were related to an increased risk for RBC transfusion after ICU stay. We suggest that strategies to prevent transfusion should focus on homogeneous subgroups of patients and take into account post-ICU needs for RBC transfusion.

## Introduction

Anaemia is a common feature in critically ill patients. In the recent ABC study [1], haemoglobin level at admission was below the normal range in 63% of patients admitted to the intensive care unit (ICU). A low haemoglobin level is associated with poor prognosis in critically ill patients [1,2], as was previously described in elderly patients with acute myocardial infarction [3].

Because anaemia commonly occurs in the ICU, red blood cell (RBC) transfusion is a frequent practice in the management of critically ill patients to compensate for acute bleeding and to increase tissue oxygen delivery [4]. Canadian and European surveys reported that up to 40% of the patients admitted to the ICU receive at least one RBC transfusion [1,5,6]. However, RBC transfusion carries short-term and long-term side effects, and liberal transfusion strategies have been associated with a worse outcome in ICU patients [6]. In an effort to avoid unnecessary RBC transfusion, intensivists have defined haemoglobin thresholds above which transfusion appears harmful [7-9]. They also proposed the use of erythropoietin [10] to avoid RBC transfusion. Although these measures may decrease blood transfusion in the ICU, they could have the opposite effect on need for transfusion after the ICU stay. Indeed, anaemia often persists or worsens after ICU discharge [1]. The ABC study [1] clearly identified the frequent need for post-ICU RBC transfusion, because 12.7% of patients who enrolled needed RBC transfusion after their ICU stay. Whether efforts to limit blood transfusion in the ICU just delay administration of RBC to the post-ICU period is unclear. In addition, although predictive factors for the need for RBC transfusion in the ICU have been identified [1,6,11], risk factors for transfusion after ICU discharge are unknown. We conducted this prospective monocentric observational study to identify risk factors for RBC transfusion in critically ill patients after discharge from the ICU.

## Materials and methods

### Patients and method

After approval had been granted by our institutional ethics committee and once informed consent had been given, we enrolled every patient admitted to our medical ICU between 1 June 2003 and 1 December 2003. The following factors were recorded for each patient on admission to the ICU: age, sex, haemoglobin level, Simplified Acute Physiology Score II [12] and Logistic Organ Dysfunction (LOD) score [13], past medical history (pulmonary disease, malignancy, cardiac disease, diabetes mellitus, thromboembolic disease, significant renal disease, haematological disorder) and cause of admission to the ICU. The use of mechanical ventilation, noninvasive ventilation, vasoactive drugs (adrenaline [epinephrine], noradrenaline [norepinephrine], dobutamine, dopamine above 5 μg/kg per min), renal replacement therapy, erythropoietin and transfusion of RBCs were also recorded, as was the length of the ICU stay and ICU outcome. All patients received standard critical care, and the decision regarding transfusion of RBCs was left to the judgement of the responsible physician. Finally, transfusion of RBCs within 7 days after ICU discharge, in-hospital length of stay following ICU discharge, and hospital outcome were also recorded. Patients were followed up until hospital discharge.

### Statistical analysis

Categorical variables are presented as values (percentage) and continuous variables as mean ± standard deviation. The odds ratios (OR), 95% confidence intervals (CI) and *P *values were calculated with exact tests for categorical data. We performed χ^2 ^tests or, when appropriate, Fisher's exact tests to assess differences between proportions with calculations of ORs and exact 95% CI. A *P *value below 0.05 was considered statistically significant.

We examined the characteristics of patients discharged from the ICU, and investigated their association with transfusion of RBCs within 7 days. We compared patients who required transfusion of RBCs (group I) with those who did not receive any transfusion within 1 week after discharge (group II). Comparison between these two groups was performed with Student's *t*-test or χ^2 ^analysis, as appropriate. Variables significantly associated with the use of transfusion of RBCs were incorporated into a stepwise logistic regression model in which the transfusion of RBC within a week after ICU discharge was the dependent outcome. The model was refined by means of stepwise selection in which a *P *value below 0.001 was used as a criterion for inclusion in the model and a *P *value above 0.01 was used as the threshold for removal from the model.

## Results

During the study period, we enrolled 550 consecutive patients who were admitted to our ICU (Table 1). Most patients (90.2%) were admitted for medical diagnosis, whereas the remaining patients were admitted for emergency (7.3%) and elective (2.5%) surgery. The overall mortality rate was 22% (122 patients). Mean haemoglobin level on admission was 11.4 ± 2.5 g/dl. Twenty per cent of the population received RBCs during their stay in the ICU. Mean haemoglobin at discharge was 10.3 ± 2.3 g/dl. Forty-seven out of the 428 patients discharged from the ICU received RBC transfusion within 1 week after discharge (group I) whereas 381 remained free from transfusion at 1 week (group II). Hospital mortality rates did not differ between the two groups.

Characteristics that differed between the groups are summarized in Table 2. Patients from group I were older and predominantly female. Patients were more likely to receive transfusion after ICU discharge if they were admitted for sepsis, or had hypotension or a medical history of malignancy. Patients admitted to the ICU for a respiratory disorder or drug poisoning were significantly less transfused than others. Severity scores on admission and discharge were higher among post-ICU transfused patients (Table 2). However, patients with haemodynamic instability requiring vasopressors surprisingly required less RBC transfusion. The mean haemoglobin level at admission in patients who received RBC transfusion within a week after ICU discharge was 8.6 g/dl. Haemoglobin level on admission and, as expected, at discharge from ICU was lower among post-ICU transfused patients.

We performed a multiple logistic regression analysis to determine variables independently associated with increased risk for RBC transfusion after ICU discharge (Table 3). Admission for sepsis (OR 341.60, 95% CI 20.35–5734.51), presence of an underlying malignancy (OR 32.6, 95% CI 3.8–280.1), female sex (OR 5.4, 95% CI 1.2–24.9), LOD score at ICU discharge (OR 1.45, 95% CI 1.1–1.9) and age (OR 1.06, 95% CI 1.02–1.12) were independently associated with RBC transfusion after ICU stay. The use of vasopressors (OR 0.01, 95%CI 0.001–0.17) and haemoglobin level at discharge from the ICU (OR 0.02, 95% CI 0.007–0.09; *P *< 0.001) were strong independent predictors of transfusion of RBCs 1 week after ICU discharge.

## Discussion

We performed the present prospective study specifically to evaluate the need for RBC transfusion during the post-ICU period. We observed that 9% of critically ill patients treated in a medical ICU required RBC transfusion after ICU discharge, and that few parameters influenced need for transfusion of RBCs within 7 days after the ICU stay.

Of patients discharged from the ICU, 11% (8.5% of the whole cohort) required RBC transfusion after ICU discharge. This is consistent with the findings of the ABC study [1], in which RBC transfusion after ICU discharge occurred in 12.7% of the population. In the present study, a considerable proportion of the patients were surgical, and this might have influenced the need for RBC transfusion.

Our study revealed that only few parameters influenced the risk for transfusion of RBCs after ICU discharge. Haemoglobin level at admission is a well established risk factor for transfusion of RBCs during the ICU stay [1,9]. We found that haemoglobin level on ICU admission was inversely correlated with the risk for transfusion in univariate analysis but not in the multivariate model. Conversely, haemoglobin level at ICU discharge markedly influenced requirement for RBC transfusion during the 7 days following ICU discharge. In our study, a 1 g/dl decrease in haemoglobin level increased by 50-fold the risk for RBC transfusion during the post-ICU stay. Previous studies demonstrated that age was strongly associated with anaemia in the critically ill. In the ABC study [1] the mean haemoglobin level at admission was significantly lower in patients older than 90 years than in patients younger than 50 years (9.9 g/dl versus 11.7 g/dl). In addition, older patients received more transfusions. We also observed a 1.06-fold increase in the likelihood of RBC transfusion for each additional year. This could be explained by an increased incidence of co-morbidities. Indeed, elderly patients frequently present with coronary artery diseases for which haemoglobin threshold values for transfusion are not clearly defined [9,14]. However, neither a previous medical history of heart disease nor cardiac disorder as the cause of admission emerged as a risk factor in our analysis. Conversely, we found that the presence of an underlying malignancy was an independent risk factor for RBC transfusion after ICU discharge. Solid neoplastic diseases occur frequently in the elderly. It is well known that they are responsible for anaemia and that their specific treatments have myelotoxic effects. The ABC study [1] also found a decreased haemoglobin level in patients who had a previous history of anaemia, especially in the setting of neoplastic disorder.

One of the most important factors associated with post-ICU transfusion of RBC was sepsis as an admission diagnosis. A previous study reported that septic patients had decreased haemoglobin levels as compared with the remainder of the ICU population [11]. Sepsis could impair production of erythropoietin by several mechanisms, including release of proinflammatory mediators that negatively impact erythropoiesis [6,15]. We recently reported that sepsis can induce anaemia by increased apoptosis of bone marrow erythroid progenitors [16]. The severity of sepsis could also lead to a greater volume of blood sampling for laboratory analysis in these patients [11].

Sepsis is frequently associated with organ failure. Whereas severity scores at admission (Simplified Acute Physiology Score II and LOD score) were reported as risk factors for ICU transfusion, they did not influence the need for transfusion after the ICU stay. On the other hand, a higher LOD score at ICU discharge was related to increased risk for RBC transfusion after the ICU stay (OR = 1.45 for each additional LOD point). Patients with persisting organ dysfunctions on ICU discharge more frequently required RBC transfusion during the remainder of their hospital stay. Surprisingly, we observed that use of vasopressors decreased the risk for RBC transfusion. The reasons for this finding are unclear. No patient had haemodynamic instability or was receiving ongoing vasopressor therapy at ICU discharge.

Our study has some limitations. First, no specific guidelines regarding RBC transfusion were given to the physicians involved in patient care after the ICU stay. Variations in the transfusion thresholds as well as in iron and vitamin supplementation policies in the various medical wards might have affected our results. Second, we limited the evaluation period to the first 7 days following ICU discharge to ascertain whether RBC requirement was directly related to the ICU stay. This delay was chosen bearing in mind the natural history of haematological disorders and the time course of myelotoxicity of drugs used in the ICU. Although we acknowledge that the validity of a 7-day period of observation is debatable, selection of the optimal follow-up period remains difficult because no study has specifically adressed this issue.

## Conclusion

Our study suggests that sepsis, underlying conditions, unresolved organ failures and haemoglobin level at discharge are related to a increased risk for RBC transfusion after ICU stay. Most of these findings are consistent with previous studies that addressed the risk for transfusion in the ICU. These findings should be considered when defining transfusion guidelines, because a higher haemoglobin level may be required in specific subgroups of ICU patients. We suggest that any strategy to prevent transfusion in the ICU should focus on homogeneous subgroups of patients and take into account post-ICU needs for RBC transfusion.

## Key messages

• Nine per cent of critically ill patients treated in a medical ICU require RBC transfusion after ICU discharge when strict transfusion guidelines are applied in the medical ICU.

• Sepsis, underlying conditions, unresolved organ failures and haemoglobin level at discharge constitute risk factors for RBC transfusion after ICU stay.

## Abbreviations

CI = confidence interval; ICU = intensive care unit; LOD = Logistic Organ Dysfunction; OR = odds ratio; RBC = red blood cell.

## Competing interests

The authors declare that they have no competing interests.

## Authors' contributions

Sophie Marque, Alain Cariou, Jean-Daniel Chiche and Yann-Erick Claessens contributed to the design of the study and drafted the manuscript. Vincent Olivier Mallet, Frédéric Pene, Jean-Paul Mira and Jean-François Dhainaut obtained the data. Sophie Marque, Alain Cariou and Yann-Erick Claessens participated in the data analysis and interpretation of the results.

## Figures and Tables

**Table 1:** Patients' characteristics and outcome

Characteristic/outcome	Value/details
Male	313 (56.9%)
Age (years)	52 (18.5)
SAPS II	39.7 ± 20.6
LOD score at ICU admission	4.2 ± 3.4
Medical history	
Cardiac disease	131 (23.8%)
Pulmonary disease	130 (23.6%)
Diabetes	49 (8.9%)
Hypertension	76 (13.8%)
Malignancy	64 (11.6%)
Thromboembolic disease	28 (5.1%)
Renal disease	46 (8.4%)
Haematologic disorder	46 (8.4%)
Purpose of admission	
Cardiac disorder	95 (17.3%)
Neurological disorder	93 (16.9%)
Respiratory disorder	140 (25.5%)
Poisoning	52 (9.5%)
Sepsis	79 (14.4%)
Haemorrhage	38 (6.9%)
Miscellanous	59 (10.7%)
Haemoglobin level on admission (g/dl)	11.4 ± 2.5
Treatment provided in the ICU	
Mechanical ventilation	304 (55.3%)
Noninvasive ventilation	188 (34.2%)
Renal replacement therapy	86 (15.6%)
Vasoactive drug	188 (34.2%)
Erythropoietin	3 (0.5%)
ICU RBC transfusion	111 (20%)
Haemoglobin level on ICU discharge (g/dl)	10.6 ± 2.3
LOD score on ICU discharge	4 ± 6.4
RBC transfusion within 7 days after ICU discharge	47 (9%)
Mortality	
ICU	122 (22%)
In-hospital	154 (28%)
Length of stay	
ICU	6.07 ± 6.93
In-hospital	14.9 ± 12.15

Results are expressed as number (percentage) or mean ± standard deviation. ICU, intensive care unit; LOD, Logistic Organ Dysfunction; RBC, red blood cell; SAPS, Simplified Acute Physiology Score.

**Table 2:** Main differences between and survival rates in patients discharged from the ICU

	RBC transfusion (*n *= 47)	No RBC transfusion (*n *= 381)	*P *value^a^
Female	33 (70%)	174 (46%)	0.038
Age (years)	55.3 ± 18.9	50.4 ± 18.4	0.021
Medical history			
Respiratory disease	5 (11%)	107 (28%)	0.010
Hypertension	11 (23%)	48 (13%)	0.043
Malignancy	13 (28%)	35 (9%)	<0.001
Diagnosis of admission			
Pulmonary disease	1 (2)	115 (28)	<0.001
Poisoning	0 (0)	49 (13)	0.009
Sepsis	18 (38)	43 (11)	<0.001
SAPS II	40.1 ± 11	33.2 ± 16.1	0.004
LOD score on admission	4.0 ± 2.3	3.1 ± 2.8	0.047
LOD score at ICU discharge	1.7 ± 1.5	0.6 ± 1.6	<0.001
Treatment provided in the ICU			
Vasoactive drugs	18 (38)	89 (23)	0.026
Haemoglobin level on admission (g/dl)	8.6 ± 1.2	11.8 ± 2.4	<0.001
Haemoglobin level at ICU discharge (g/dl)	7.5 ± 0.8	11.2 ± 2.0	<0.001
Length of stay in the ICU (days)	4.8 ± 3.0	5.4 ± 6.2	0.047
In-hospital outcome			
Alive	43 (91)	353 (93)	0.56

Data are expressed as number (percentage) or mean ± standard deviation. ^a^Dependent two-tailed *t*-test. ICU, intensive care unit; LOD, Logistic Organ Dysfunction; SAPS, Simplified Acute Physiology Score.

**Table 3:** Multiple logistic regression analysis of predictive factors for RBC transfusion within 7 days after ICU discharge

Parameters	OR (95% CI)
Sepsis	341.6 (20.3–5734.5)
Medical history of malignancy	32.6 (3.8–280.1)
Female sex	5.4 (1.2–24.9)
LOD score at ICU discharge^a^	1.45 (1.1–1.9)
Age^b^	1.06 (1.02–1.12)
Vasopressors	0.01 (0.001–0.17)
Haemoglobin level on ICU discharge^c^	0.02 (0.007–0.09)

^a^OR adjusted for 1 point.^b^OR adjusted for 1 year. ^c^OR adjusted for 1 g/dl. CI, confidence interval; ICU, intensive care unit; LOD, Logistic Organ Dysfunction; OR, odds ratio; RBC, red blood cell.
